# Maximal violation of Bell inequalities under local filtering

**DOI:** 10.1038/srep46505

**Published:** 2017-04-18

**Authors:** Ming Li, Huihui Qin, Jing Wang, Shao-Ming Fei, Chang-Pu Sun

**Affiliations:** 1 College of the Science, China University of Petroleum, Qingdao 266580, P. R. China; 2 School of Mathematics and Statistics, Hainan Normal University, Haikou 571158, P. R. China; 3 Max-Planck-Institute for Mathematics in the Sciences, Leipzig 04103, Germany; 4 School of Mathematical Sciences, Capital Normal University, Beijing 100048, P. R. China; 5 Beijing Computational Science Research Center, Beijing 100048, P. R. China

## Abstract

We investigate the behavior of the maximal violations of the CHSH inequality and V*è*rtesi’s inequality under the local filtering operations. An analytical method has been presented for general two-qubit systems to compute the maximal violation of the CHSH inequality and the lower bound of the maximal violation of V*é*rtesi’s inequality over the local filtering operations. We show by examples that there exist quantum states whose non-locality can be revealed after local filtering operation by the V*é*rtesi’s inequality instead of the CHSH inequality.

Quantum mechanics is inherently nonlocal. After performing local measurements on a composite quantum system, non-locality, which is incompatible with local hidden variable theory^1^ can be revealed by Bell inequalities. The non-locality is of great importance both in understanding the conceptual foundations of quantum theory and in investigating quantum entanglement. It is also closely related to certain tasks in quantum information processing, such as building quantum protocols to decrease communication complexity^2^,^3^ and providing secure quantum communication^4^,^5^. We refer to ref. 6 for more details.

To determine whether a quantum state has non-locality, it is sufficient to construct a Bell inequality^7^,^8^,^9^,^10^,^11^,^12^,^13^ which can be violated by the quantum state. For two qubits systems, Clauser-Horne-Shimony-Holt have presented the famous CHSH inequality^7^.

Let 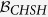 denote the Bell operator for the CHSH inequality,


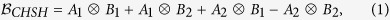


with *A*_*i*_ and *B*_*j*_ being the observables of the form 
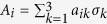
 and 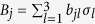 respectively, *i, j* = 1, 2,


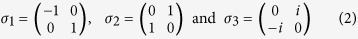


are the Pauli matrices. For any two-qubit quantum state *ρ*, the maximal violation of the CHSH inequality (MVCI) is given by^14^


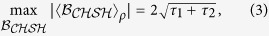


where *τ*_1_ and *τ*_2_ are the two largest eigenvalues of the matrix *T*^†^*T, T* is the matrix with entries 
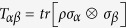
, *α, β* = 1, 2, 3, † stands for transpose and conjugation. For a state admitting local hidden variable (LHV) model, one has 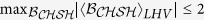.

Another effective Bell inequality for two-qubit system is given by the Bell operator^15^ V*é*rtesi


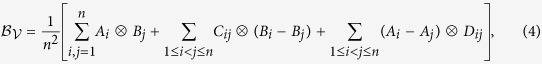


where *A*_*i*_, *B*_*j*_, *C*_*ij*_ and *D*_*ij*_ are observables of the form 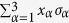 with 
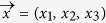
 the unit vectors.

The maximal violation of V*é*rtesi’s inequality(MVVI) is lower bounded by the following inequality^16^. For arbitrary two-qubit quantum state *ρ*, we have


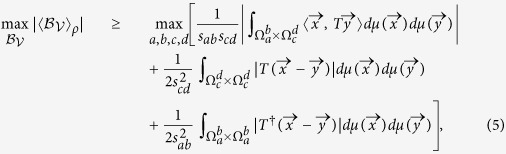


where 
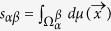
. The maximum on the right side of the inequality goes over all the integral area 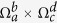 with 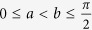 and 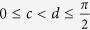. Here the maximal value 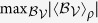 of a state *ρ* admitting LHV model is upper bounded by 1.

The maximal violation of a Bell inequality above is derived by optimizing the observables for a given quantum state. With the formulas (3) and (5) one can directly check if a two-qubit quantum state violates the CHSH or the V*é*rtesi’s inequality. It has been shown that the maximal violation of a Bell inequality is in a close relation with the fidelity of the quantum teleportation^17^ and the device-independent security of quantum cryptography^18^.

The maximal violation of a Bell inequality can be enhanced by local filtering operations^19^. In ref. 20, the authors present a class of two-qubit entangled states admitting local hidden variable models, and show that the states after local filtering violate a Bell inequality. Hence, there exist entangled states, the non-locality of which can be revealed by using a sequence of measurements.

In this manuscript, we investigate the behavior of the maximal violations of the CHSH inequality and V*é*rtesi’s inequality under local filtering operations. An analytical method has been presented for any two-qubit system to compute the maximal violation of the CHSH inequality and the lower bound of the maximal violation of V*é*rtesi’s inequality under local filtering operations. The corresponding optimal local filtering operation is derived. We show by examples that there exist quantum states whose nonlocality can be revealed after local filtering operation by V*é*rtesi’s inequality instead of the CHSH inequality.

## Results

We consider the CHSH inequality for two-qubit systems first. Before the Bell test, we apply the local filtering operation on a state 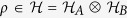 with 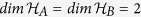. *ρ* is mapped to the following form under local filtering transformations^20^,^21^:


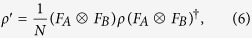


where 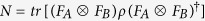 is a normalization factor, and *F*_*A/B*_ are positive operators acting on the subsystems respectively. Such operations can be a local interaction with the dichroic environments^22^.

For two-qubit systems, let 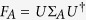 and 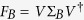 be the spectral decompositions of *F*_*A*_ and *F*_*B*_ respectively, where *U* and *V* are unitary operators. Define that


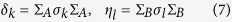


and *X* be a matrix with entries given by


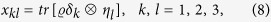


where 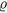 is locally unitary with *ρ*.

We have the following theorem.

**Theorem 1:** The maximal quantum bound of a two-qubit quantum state 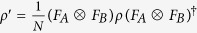 is given by


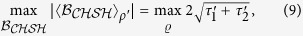


where 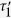 and 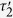 are the two largest eigenvalues of the matrix *X*^†^*X/N*^2^ with *X* given by (8). The left max is taken over all *B*_*CHSH*_ operators, while the right max is taken over all 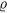 that are locally unitary equivalent to *ρ*.

See Methods for the proof of theorem 1.

Now we investigate the behavior of the V*è*rtesi-Bell inequality under local filtering operations. In ref. 16 we have found an effective lower bound for the MVVI by considering infinite many measurements settings, *n* → ∞. Then the discrete summation in (4) is transformed into an integral of the spherical coordinates over the sphere 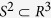. We denote the spherical coordinate of *S*^2^ by (*ϕ*_1_, *ϕ*_2_). A unit vector 
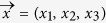
 can be parameterized by *x*_1_ = sin *ϕ*_1_ sin *ϕ*_2_, *x*_2_ = sin *ϕ*_1_ cos *ϕ*_2_, *x*_3_ = cos *ϕ*_1_. For any 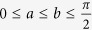, we denote 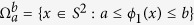.

**Theorem 2:** For two-qubit quantum state *ρ*′ given by (6), we have


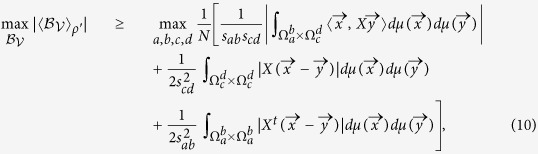


where *X* is defined by (8). *X*^*t*^ stands for the transposition of *X*, and 
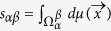
. The maximization on the right side of the inequality goes over all the integral area 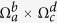 with 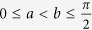 and 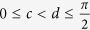.

See Methods for the proof of theorem 2.

**Remark:** The right hand sides of (9) and (10) depend just on the state *σ* which is local unitary equivalent to *ρ*. Thus to compare the difference of the maximal violation for *ρ* and that for *ρ*′, it is sufficient to just consider the difference between *σ* and *ρ*′.

Without loss of generality, we set


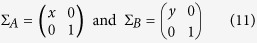


with *x, y* ≥ 0. According to the definition of *δ*_*k*_ and *η*_*l*_ in (7), one computes that


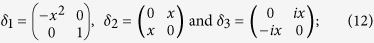



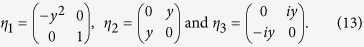


Let 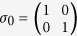. Set 
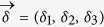
, 
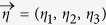
, and 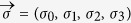. We have 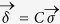 and 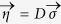, where


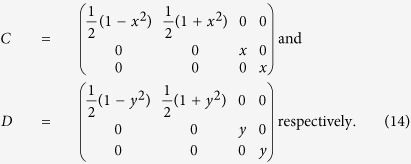


Then one has *x*_*kl*_ = (*CWD*^†^), where *W* is a 4 × 4 matrix with entries 
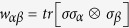
. Let 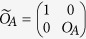 and 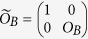 where *O*_*A*_ and *O*_*B*_ are 3 × 3 orthogonal operators. Define that 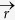 and 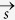 be three dimensional vectors with entries 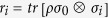 and 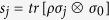 respectively. And let 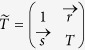. One can further show that


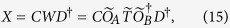


and


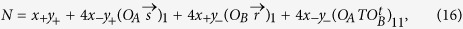


where 
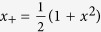
, 
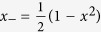
, 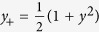 and 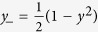. Numerically, one can parameterize *O*_*A*_ and *O*_*B*_ and then search for the maximization in theorem 1. For the lower bound in theorem 2, we refer to ref. 16.

**Corollary:** For two-qubit Werner state^23^ 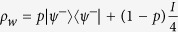, with 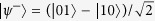, one computes 
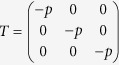
. Then by using the symmetric property of the state, (15) and (16), together with theorem 1, we have


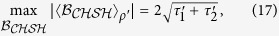


where 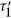 and 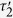 are the two largest eigenvalues of the matrix *X*^†^*X/N*^2^ with *X* given by


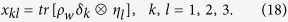


## Applications

In the following we discuss the applications of local filtering. First we show that a state which does not violate the CHSH and the V*é*rtesi’s inequalities could violate these inequalities after local filtering. Consider the following density matrix for two-qubit systems:


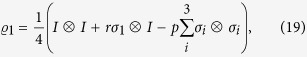


where −0.3104 ≤ *p* ≤ 0.7 to ensure the positivity of 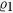. By using the positive partial transposition criteria one has that 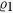 is separable for −0.3104 ≤ *p* ≤ 0.3104.

**Figure 1:**
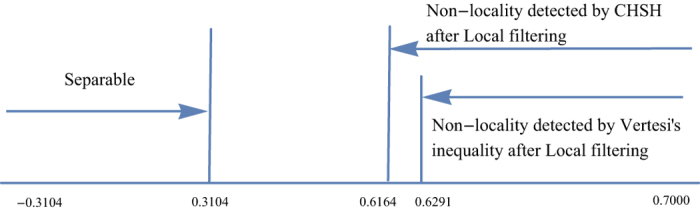
For *r* = 0.3, both the CHSH inequality and Vértesi’s inequality fail to detect the non-locality of 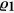 for the whole parameter region of *p*. After local filtering, non-locality is detected for 0.6291 ≤ *p* ≤ 0.7 (by Theorem 2) and 0.6164 ≤ *p* ≤ 0.7 (by Theorem 1) respectively.

Case 1: Set *r* = 0.3. It is direct to verify that both the CHSH inequality and V*é*rtesi’s inequalities fail to detect the non-locality for the whole region −0.3104 ≤ *p* ≤ 0.7. After filtering, non-locality can be detected for 0.6291 ≤ *p* ≤ 0.7 (by Theorem 2) and 0.6164 ≤ *p* ≤ 0.7 (by Theorem 1) respectively, see Fig. 1.

Case 2: Set *p* = 0.7050 and *r* = 0.0400. The MVCI of 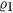 is 1.994 without local filtering and 1.9988 after local filtering, which means that the CHSH inequality is always satisfied before and after local filtering. The lower bound (5) for 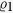 is computed to be less than one, implying the non-locality can not be detected by the lower bound for MVVI derived in ref. 16 without local filtering. However, by taking *x* = *y* = 1.1, *a* = *c* = 0.1671, *b* = *d* = 1.1096, from Theorem 2 we have the maximal violation value 1.0005 which is larger than one. Therefore, after local filtering the state’s non-locality is detected.

Next we give an example that a state admits local hidden variable model (LHV) can violate the Bell inequality under local filtering. Consider two-qubit quantum states with density matrices of the following form:


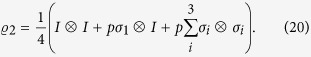


According to the positivity of a density matrix, we have −0.5 ≤ *p* ≤ 0.3090. By using the positive partial transposition criteria^24^, one checks that 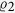 is entangled for −0.5 ≤ *p* ≤ 0.3090. The quantum state satisfies the CHSH inequality for the whole parameter region.

We first show that the state 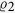 admits LHV models for −0.5 ≤ *p* ≤ −0.3090.

First we rewrite 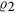 as a convex combination of singlet and separable states,


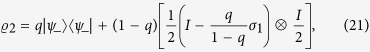


where 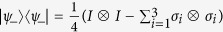 and *q* = −*p*. According to ref. 25, with a visibility of 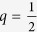, the correlations of measurement outcomes produced by measuring the observables 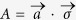 and 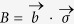 on the singlet state can be simulated by an LHV model in which the hidden variable 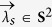 is biased distributed with probability density


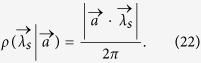


With probability 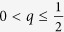, Alice and Bob can share the biased distributed variable resource and output 
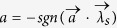
 and 
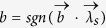
, respectively. With probability 1 − *q*, Alice outputs *a* = ±1 with probability 
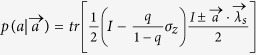
, and Bob outputs ±1 with probability 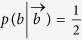. Then we can simulate the correlations produced by measuring obesrvables *A* and *B* on 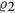,


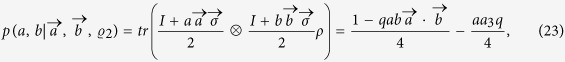


which can be given by the following LHV model,


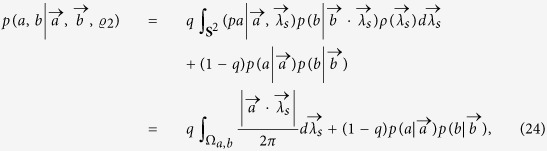


where 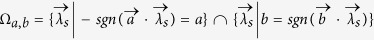. Explicitly,


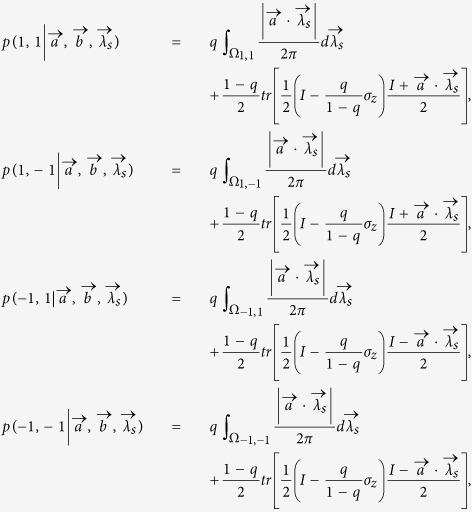


where 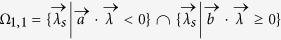, 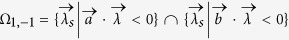, 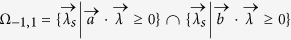, 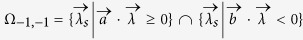.

**Figure 2:**
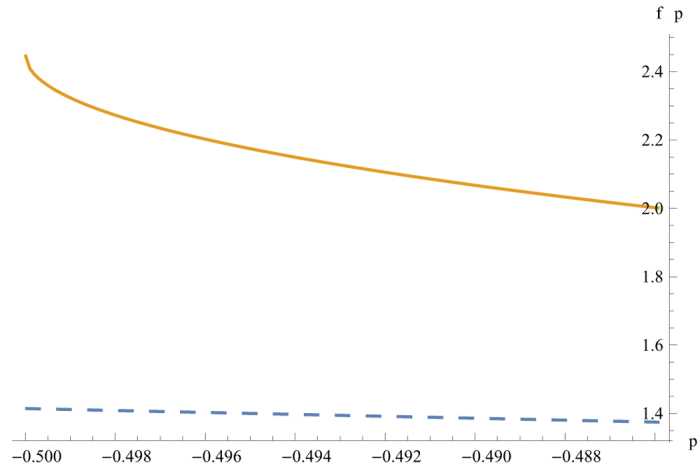
The MVCI of 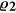 (dashed line) v.s. the MVCI after Local filtering (solid line). *f(p)* stands for the MVCI. Note that the classical bound of the CHSH inequality is 2.

Therefore the state 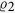 admits LHV model for −0.5 ≤ *p* ≤ −0.309. However, after local filtering, non-locality (violation of the CHSH inequality) is detected for −0.5 ≤ *p* ≤ −0.4859, see Fig. 2.

**Remark:** In ref. 17 Horodeckis have presented the connection between the maximal violation of the CHSH inequality and the optimal quantum teleportation fidelity:


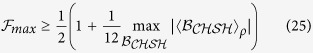


which means that any two-qubit quantum state violating the CHSH inequality is useful for teleportation and vice versa. Ac*í*n *et al*. have derived the relation between the maximal violation of the CHSH inequality and the Holevo quantity between Eve and Bob in device-independent Quantum key distribution (QKD)^18^:


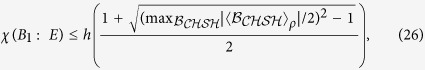


where *h* is the binary entropy. From our theorem, 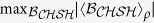 can be enhanced by implementing a proper local filtering operation from smaller to larger than 2, which makes a teleportation possible from impossible, or can be improved to obtain a better teleportation fidelity. The proper (optimal) local filtering operation can be selected by the optimizing process in (9) together with the double cover relationship between the *SU*(2) and *SO*(3). For application in the QKD, Eve can enhance the upper bound of Holevo quantity by local filtering operations which makes a chance for attacking the protocol.

## Discussions

It is a fundamental problem in quantum theory to recognize and explore the non-locality of a quantum system. The Bell inequalities and their maximal violations supply powerful ability to detect and qualify the non-locality. Furthermore, the constructing and the computation of the maximal violation of a Bell inequality is in close relationship with quantum games, minimal Hilbert space dimension and dimension witnesses, as well as quantum communications such as communication complexity, quantum cryptography, device-independent quantum key distribution etc. ref. 6. A proper local filtering operation can generate and enhance the non-locality. We have investigated the behavior of the maximal violations of the CHSH inequality and the V*é*rtesi’s inequality under local filtering. We have presented an analytical method for any two-qubit system to compute the maximal violation of the CHSH inequality and the lower bound of the maximal violation of V*é*rtesi’s inequality under local filtering. We have shown by examples that there exist quantum states whose nonlocality can be revealed by local filtering operations in terms of the V*é*rtesi’s inequality instead of the CHSH inequality.

## Methods

### Proof of Theorem 1 and Theorem 2

The normalization factor *N* has the following form,


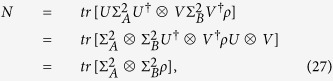


where 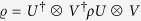. Since *ρ* and 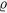 are local unitary equivalent, they must have the same value of the maximal violation for CHSH inequality.

We have that


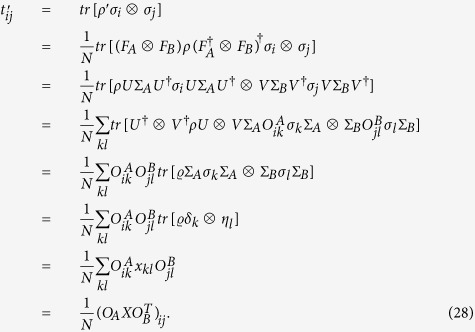


In deriving the fourth equality in (28) we have used the double cover relation between the special unitary group *SU*(2) and the special orthogonal group *SO*(3): for any given unitary operator *U*, 
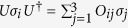
, where the matrix *O* with entries *O*_*ij*_ belongs to *SO*(3)^26^,^27^.

Finally, one has that


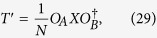


and


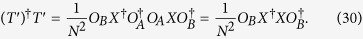


By noticing the orthogonality of the operator *O*_*B*_ we have that the eigenvalues of (*T*′)^†^*T*′ and *X*^†^*X/N*^2^ must be the same, which proves theorem 1.

We can further obtain theorem 2 by substituting (29) into (5).

## Additional Information

**How to cite this article**: Li, M. *et al*. Maximal violation of Bell inequalities under local filtering. *Sci. Rep.* **7**, 46505; doi: 10.1038/srep46505 (2017).

**Publisher's note:** Springer Nature remains neutral with regard to jurisdictional claims in published maps and institutional affiliations.
